# Incidence of catheter-associated urinary tract infections by Gram-negative bacilli and their ESBL and carbapenemase production in specialized hospitals of Bahir Dar, northwest Ethiopia

**DOI:** 10.1186/s13756-024-01368-7

**Published:** 2024-01-25

**Authors:** Zelalem Asmare, Tewachew Awoke, Chalachew Genet, Alemale Admas, Addisu Melese, Wondemagegn Mulu

**Affiliations:** 1 Department of Medical Laboratory Science, College of Health Sciences, Woldia University, Woldia, Ethiopia; 2 Department of Medical Laboratory Sciences, College of Medicine and Health Sciences, Bahir Dar University, Bahir Dar, Ethiopia

**Keywords:** CAUTIs, GNB, Patients, Chromogenic medium, FHCSH, TGSH, MDR, ESBL, Ethiopia

## Abstract

**Background:**

Catheter-associated urinary tract infections (CAUTIs) due to multidrug-resistant Gram-negative bacilli (GNB) is a common concern globally. Investigating the incidence of CAUTI and associated antibiotic resistance has paramount importance from the health care associated infections perspective. This study therefore assessed the incidence of CAUTIs due to GNB and the production of extended-spectrum beta-lactamase (ESBL) and carbapenemase among inpatients in specialized hospitals of Northwest, Ethiopia.

**Methods:**

A total of 363 patients with indwelling urinary catheters who were admitted in the hospital for > 48 h were consecutively enrolled and followed from 3 to 18 days. Data were collected through interviewing and review of medical records. Patients who developed at least one of the following: fever (> 38 ^O^C), suprapubic tenderness, or costovertebral angle pain, coupled with a GNB positive urine culture of ≥ 10^3^ CFU/mL with no more than two bacterial species were defined as CAUTI. The ESBL and carbapenemase production were detected and identified by chromogenic medium. Logistic regression analysis was done to identify associated factors.

**Results:**

From 363 patients followed, the incidence rate of CAUTI was 27.8 per 1000 catheter days. Catheterization for ≥ 8 days (AOR = 10.6, 95%CI:1.8–62.1) and hospitalization for > 10 days (AOR = 8.1, 95%CI: 2.4–27.2) were the factors significantly associated with CAUTIs. *E. coli* (*n* = 18, 34.6%), *Proteus* species (*n* = 7, 13.5%), and *P. aeruginosa* (*n* = 6, 11.5%) were the most frequent GNB. Isolates revealed high rates of resistance to amoxicillin-clavulanic acid (100%), cefazolin (*n* = 51, 98%), ceftazidime (*n* = 47, 90%) and cefotaxime (*n* = 46, 88%). Most of the GNB isolates (86.5%) were multidrug-resistant. Overall, 19.2% and 5.8% of GNB isolates were ESBL and carbapenemase producers, respectively.

**Conclusions:**

Incidence of CAUTI with Gram-negative bacilli is high. As most of the GNB isolates are MDR and showed a super high rate of resistance to amoxicillin-clavulanic and third-generation cephalosporins, empirical treatment with these substances is virtually ineffective in patients with suspected GNB infection in Ethiopia. The expression of ESBL and carbapenemase among GNB isolates is also a concern. Therefore, improved infection prevention and control measures, careful use of catheters and third generation of cephalosporins are needed to improve patient outcomes and reduce the burden of CAUTIs and the spreading of antimicrobial resistance.

## Background

Catheter-associated urinary tract infections (CAUTIs) account for 70–80% of nosocomial urinary tract infections (UTIs) and are responsible for a high burden of morbidity and mortality in hospitalized patients [1]. They are serious public health issues, with the consequences including prolonged hospitalizations, long-term disability, increasing antimicrobial resistance (AMR), additional financial burden for the healthcare system, high patient expenses, and adverse patient outcomes [2, 3]. In the United States, an estimated 449,334 CAUTIs occur per year [4]. The burden of CAUTI is higher in lower-income countries as compared to countries in Europe and the United States of America (USA). For instance, a systematic review across five regions of WHO reported 4.4, 9.5, 14.7, 9.5, 10, and 7.2 CAUTI incidence rates per 1000 catheter days in USA, Europe, Southeast Asia, Eastern Mediterranean, and Western Pacific, respectively [5]. In Ethiopia, two studies documented an incidence rate of 28.1 and 60.2 CAUTIs per 1000 catheter days, respectively [6, 7].

Gram-negative bacilli (GNB), such as *Escherichia* (*E*.) *coli*, *Klebsiella* (*K*.) *pneumoniae*, *Pseudomonas* (*P*.) *aeruginosa, Proteus mirabilis*, *and Acinetobacter* (*A*.) *baumannii*, are the most common pathogens involved in CAUTIs [8]. Recent studies from around the world have reported that *K*. *pneumoniae*, *A. baumannii*, *E. coli*, and *P. aeruginosa* are involved in 13.3–53.3%, 8–25%,18.5–40.5%, and 7.1–20.2% of CAUTI, respectively [6, 9–14]. Beta-lactam antibiotics, including third-generation cephalosporins, are commonly used for the treatment of UTIs caused by GNB currently [15]. Carbapenems are one of the few options for the treatment of multidrug-resistance (MDR) pathogens, including ESBL producing bacteria [16, 17]. Gram-negative bacilli isolates resistant to at least one antibiotic in three or more antibiotic classes are MDR [18].

Antimicrobial resistance is a global public health threat, causing the emergence and spread of multi-and pan-drug-resistant bacteria [19]. Misuse and overuse of antimicrobials are the main causes [19]. Extended-spectrum beta-lactamase (ESBL) enzyme production is a common antibiotic resistance mechanisms in GNB [15]. Nowadays, carbapenemase production has emerged as a significant drug resistance mechanism. There is evidence that the spread of MDR bacteria in Ethiopia is leading to a worrying increase in mortality rates from various bacterial infections locally [15, 20].

Despite global estimates reported that low-income countries have a higher CAUTI burden than high-income countries, there is a scarcity of data on the actual burden of CAUTI in sub-Saharan African countries. Gram-negative bacilli isolated from various clinical specimens show a high resistance rate to commonly used antibiotics in low-income countries, including Ethiopia. However, epidemiological information on ESBL and carbapenemase production among patients with CAUTI remains insufficient. In Ethiopia, previous research on ESBL primarily overlooked CAUTIs. The 49% and 41.2% reported pooled rates of ESBL production in Enterobacteriaceae isolates were from various specimens and urine samples, respectively [21]. Similarly, the 2.7–8% carbapenemase production among GNB isolates reported before was not specifically from CAUTIs [20, 22–24]. Therefore, data on the incidence of CAUTIs caused by GNB and associated ESBL and carbapenemase production is very scarce in Ethiopia. The factors associated with CAUTIs were not reported. In addition, there was no report on the detection of GNB using chromogenic medium, where resistance profiles typical for ESBL or carbapenem production are fast and reliable [25–28]. As a result, there are knowledge gaps regarding the incidence of CAUTIs, ESBL, and carbapenemase production in GNB, along with associated antibiotic resistance profiles in Ethiopia.

Therefore, current and local AMR surveillance data is essential for the proper management of patients, implementation of enhanced infection prevention and control measures, and containment of the spreading of AMR. Thus, this study assessed the incidence of GNB-associated CAUTI, determined the ESBL and carbapenemase expression rates of GNB, and identified the associated factors of CAUTI among patients attending specialized hospitals in Bahir Dar, northwest Ethiopia.

## Methods

### Study design, setting and period

A longitudinal study was carried out between January and July 2022 at Felege Hiwot Comprehensive Specialized Hospital (FHCSH) and Tibebe Ghion Specialized Hospital (TGSH) in Bahir Dar, Ethiopia. These hospitals are among the largest specialized hospitals in Ethiopia, serving a population of over 7 million people. FHCSH has 12 wards with 500 beds and 1416 healthcare staff, while TGSH has 13 wards with 493 beds. On average, TGSH and FHCSH admit 13 and 12 catheterized patients per day, respectively.

### Sample size and sampling

The sample size was calculated using a single population proportion formula, taking into account 5% margin of error and a 61.7% of ESBL production in Enterobacteriaceae isolated from UTI in a previous research finding [29]. The calculated 363 was taken as the final sample size and was allocated proportionally to TGSH and FHCSH based on their daily catheterized patient intake. Consequently, the study included and followed 174 patients from FHCSH and 189 patients from TGSH. All consenting catheterized patients meeting the inclusion criteria were consecutively enrolled until the required sample size was reached.

### Variables

Incidence of CAUTI was the outcome variable, while socio-demographic characteristics (age, sex, occupation, residence, and level of education), presence of underlying disease, and hospitalization-related variables (such as the ward to which the patient was admitted, history of catheterization, previous hospitalization within the last 12 months, days of catheterization, and days of hospitalization) were the independent variables.

### Study population and sampling technique

The study included patients from FHCSH and TGSH ICU, surgical, medical, and gynecological wards who had an indwelling urinary catheter for over two days and were willing to participate. These patients were followed by internists for the development of features suggestive of CAUTI until catheters were removed. We define CAUTI as the presence of at least one of the clinical signs and symptoms: fever (> 38 ^O^C), suprapubic tenderness, costovertebral angle pain or tenderness in a patient that had been in place for more than two consecutive days in an inpatient location and a positive urine culture of ≥ 10^3^ with no more than two bacterial species [30]. Socio-demographic characteristics and clinical information of each study participant were collected through face-to-face interviews using questionnaires supported by a review of the patients’ medical records.

### Specimen collection

Urine specimens (10 mL) were collected only from patients symptomatic for CAUTI through the catheter port by detaching the bag from the connecting rubber to the urethra and transferred into wide-mouthed, clean, screw-capped containers [31]. After collection, specimens were transported immediately to the Microbiology Laboratory at Felege Hiwot Campus, Bahir Dar University, using an icebox, and processed immediately without any delays.

### Detection and identification of Gram-negative bacilli from urine sample

A small volume of urine specimen (0.001 mL) was directly streaked onto MacConkey Agar (Oxoid, Basingstoke, UK) using a sterile calibrated inoculating loop and incubated for 24 h at 37^o^C under normal atmospheric conditions [32]. Bacterial growths of ≥ 10^3^ CFU/mL [30] were considered significant bacteriuria, and GNB was identified following a standard protocol.

Isolates from plates with two or more visible colonies were sub-cultured onto Blood Agar plates ((Blood Agar base, HiMedia, Mumbai, India), with 5% sheep blood). The plates were incubated for 24 h under normal atmospheric conditions. Gram-negative bacilli growth was identified by their colony characteristics and biochemical tests such as sugar fermentation, H2S and gas production, citrate utilization, motility tests, indole production, urease, and oxidase tests.

### Antibiotic susceptibility testing

Susceptibility of GNB isolates to ten antibiotics such as cefazolin (30 µg), cefotaxime (30 µg), ceftazidime (30 µg), amoxicillin-clavulanic acid (20/30µg), nitrofurantoin (10 µg), trimethoprim-sulfamethoxazole (1.25/23.75 µg), ciprofloxacin (5 µg), imipenem (10 µg), and meropenem (10 µg) (Oxoid, Basingstoke, UK) and cefotaxime-clavulanate, (30/10µg) (Abtek, Liverpool, UK) was performed on Mueller Hinton agar (Condalab, Madrid, Spain) using the Kirby Bauer disk diffusion technique in accordance with the guidelines of Clinical and Laboratory Standards Institute (CLSI) (CLSI, 2021). The bacterial growth around the discs was observed, and the diameter of the zone of inhibition was measured using a caliper and interpreted according to the 2021 CLSI guideline. Isolates that showed non-susceptibility to at least one agent in three or more antibiotic classes were classified as having multi-drug resistance (MDR) [18].

### Detection and identification of ESB production

ESBL production in GNB was screened considering the zone of inhibition diameters produced by ceftazidime (30 µg) or cefotaxime (30 µg) in the disk diffusion technique following the 2021 CLSI recommendations [33]. Gram-negative bacilli isolates with zone of inhibition diameters of ≤ 27 mm for cefotaxime and ≤ 22 mm for ceftazidime were considered as likely ESBL producers. These isolates were further checked for ESBL production using a selective Chromatic TM ESBL ((Liofilchem, Province of Teramo, Italy), Chromatic TM ESBL agar base with a 1% Chromatic ESBL supplement (81,089)) [33]. Briefly, the suspected ESBL-producing isolates were suspended in normal saline, inoculated onto Chromatic ESBL agar, and incubated at 37 ^O^C for 24 h under normal atmospheric conditions. The ESBL production was considered confirmed by observing growth of specific pigmented colonies of GNB on Chromatic ESBL agar. Bacterial species were identified following the chromogenic color code according to the manufacturer’s instructions [34, 35].

### Detection and identification of carbapenemase production

The likely carbapenemase production in GNB was initially screened based on the susceptibility profiles of isolates to imipenem and meropenem. Gram-negative bacilli isolates that displayed resistance to imipenem and/or meropenem were suspected of carbapenemase producer and further checked by using a chromogenic medium called Chromatic CRE (Chromatic CRE agar base with a 1% Chromatic CRE supplement (81,088), (Liofilchem, Province of Teramo, Italy)) [33]. Briefly, two to three separate colonies of GNB isolates were suspended in 3 mL of normal saline and inoculated onto Chromatic CRE agar. Carbapenemase production was considered confirmed by observing growth of specific pigmented colonies of GNB on Chromatic CRE agar. Bacterial species were identified following the chromogenic color code according to manufacturer instructions [36].

### Quality control

Aseptic techniques were followed during urine sample collection and processing. The sterility of culture medium was checked by a sterility indicator tape. The sterility of culture media were tested by incubating 3–5% of the batch of prepared media at 37 °C for 24 h, and their performance was checked by inoculating control microorganisms. American Type Culture Collection (ATCC) standard reference strains such as *E. coli* ATCC 25,922 and *K. pneumoniae* ATCC 700,603 were used as a negative and positive control for ESBL production, respectively. *K. pneumoniae* ATCC BAA 1705 for carbapenemase production positive control and *K. pneumoniae* ATCC BAA 1706 for carbapenemase production negative control were used to ensure the reliability and accuracy of the test.

### Data analysis

Data were analyzed using IBM SPSS Statistics for Windows, version 27.0. Armonk, NY: IBM Corp., SYSTAT. Descriptive statistics were computed, and relevant variables were expressed in frequency and percentages. Both Kolmogorov-Smirnov and Shapiro-Wilk tests were calculated to check the normality of continuous data. When data were not normally distributed, the median and interquartile range (IQR) Q1 to Q3 were reported. The incidence rate of CAUTIs was calculated and expressed as the number of new CAUTI cases per 1000 catheter days followed. Logistic regression analysis was performed to analyze the association between independent variables and the incidence of CAUTIs. Variables with a *p*-value of ≤ 0.2 in the bivariable analysis were further entered into a multivariable logistic regression to identify independent predictors of CAUTI and drug resistance. Assumptions for binary logistic regression were checked, such as the Hosmer-Lemeshow goodness of fit test, the receiver operating characteristic (ROC) curve for multicollinearity check, and data were checked for the presence of outliers. *P*-value < 0.05 was taken as cut-off for statistical significance.

## Results

### Socio-demographic profile and clinical presentation of patients with urinary catheter

As shown in Table 1, a total of 363 patients participated in the study. Of which, 189 (52.1%) were from TGSH. The age of patients ranged from 6 to 92 years, with a median age of 42 years (IQR, 31–53). In terms of patient’s ward, (*n* = 135, 37.2%) were from the surgical ward, and (*n* = 114, 31.4%) were from the medical ward (Table 1).

**Table 1:** Culture confirmed catheter associated urinary tract infections caused by Gram-negative bacilli and demographic variables of study participants (*n* = 363)

Variables	Culture result			*p*-value
	Positive	Negative	Total	
	N (%)	N (%)	N (%)	
**Age in years**				
Median (IQR)	38 (32–55)	42 (30.25-57)	42 (31–53)	0.57
**Gender**				
Female	21 (41.2)	119 (38.1)	140 (38.6)	0.295
Male	30 (58.8)	193 (61.9)	223 (61.4)	
**Residence**				
Rural	33(64.7)	154 (49.4)	187 (51.5)	0.023
Urban	18 (35.3)	158 (50.6)	176 (48.5)	
**Level of Education**				
No read and write	18 (35.3)	93 (29.8)	111 (30.6)	
Primary education	21 (41.2)	106 (34)	127 (35)	0.317
Secondary education	8 (15.7)	62 (19.9)	70 (19.3)	
Diploma and above	4 (7.8)	51 (16.3)	55 (26.1)	
**Patients’ hospital**				
FHCSH	29 (56.9)	145 (46.5)	174 (47.9)	0.089
TGSH	22 (43.1)	167 (53.5)	189 (52.1)	
**Patient ward**				
Medical	7 (13.7)	107 (34.3)	114 (31.4)	
Surgical	27 (52.9)	108 (34.6)	135 (37.2)	0.013
ICU	12 (23.5)	63 (20.2)	75 (20.7)	
Gynecology	5 (9.8)	34 (10.9)	39 (10.7)	
**Total**	**51 (14)**	**312 (86)**	**363**	

**Key**: TGSH: Tibebe Ghion Specialized Hospital; FHCSH: Felege Hiwot Comprehensive Specialized Hospital; ICU: Intensive Care Unit

All 363 patients were followed for a total of 1835 catheter days, with a median duration of 5 days (IQR, 3–6). Out of these, 58.1% (*n* = 211) patients developed at least one of the following clinical signs of UTI: fever, suprapubic tenderness, and costovertebral angle pain.

### Culture-confirmed incidence of CAUTI caused by GNB

**Fig. 1:**
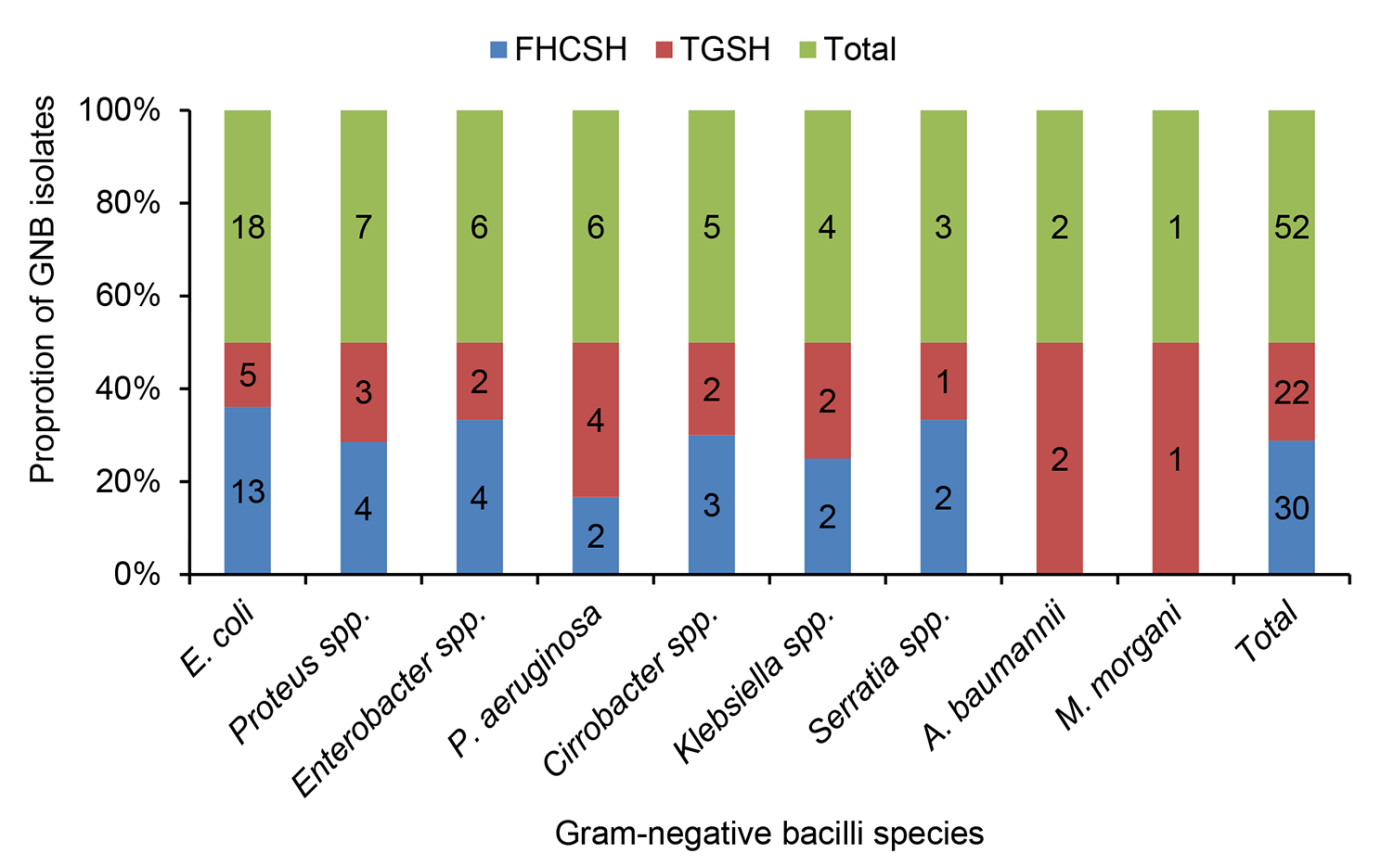
Frequency of Gram-negative bacilli species isolated from patients with catheter associated urinary tract infection GNB: Gram-negative bacilli; FHCSH- Felege Hiwot Comprehensive Specialized Hospital; TGSH- Tibebe Ghion Specialized Hospital

Out of 211 patients who developed clinical signs of CAUTI, 51 (24.2%) had a positive culture for GNB. Notably, one sample had two GNB isolates. The overall incidence rate of CAUTI caused by GNB was 27.8 per 1000 catheter days (51/1835). The proportion of GNB-caused CAUTI was significantly higher in patients from rural (64.7%) than urban (35.3%) residents (*p* = 0.023). In addition, the occurrence of GNB-caused CAUTI was significantly higher among patients from surgical wards (52.9%) compared to those from ICU (23.5%), medical (13.7%), and gynecology (9.8%) wards (*p* = 0.013) (Table 1). The proportion of GNB that caused CAUTI was 34.2 per 1000 catheter days in FHCSH and 22.3 per 1000 catheter days in TGSH. However, this difference was not statistically significant (*p* = 0.89). Of the 52 GNB isolates, 30 (57.7%) were identified in patients from FHCSH, and the remaining 22 (42.3%) were from TGSH (Fig. 1).

### Gram-negative bacilli species profile

*E. coli* was the most frequent isolate (*n* = 18, 34.6%), followed by *Proteus* species (*n* = 7, 13.5%), and *Enterobacter* species as well as *P*. *aeruginosa* (*n* = 6, 11.5%) (Fig. 1). The majority of GNB isolates (*n* = 27, 51.9%), were from patients in surgical wards, whereas all *A. baumannii* isolates were exclusively from the ICU ward. The proportion of GNB was non-significantly higher among males (58.8%) than females (41.2%) (*p* = 0.295) (Table 1).

### Proportion of ESBL and carbapenemase expression

**Fig. 2:**
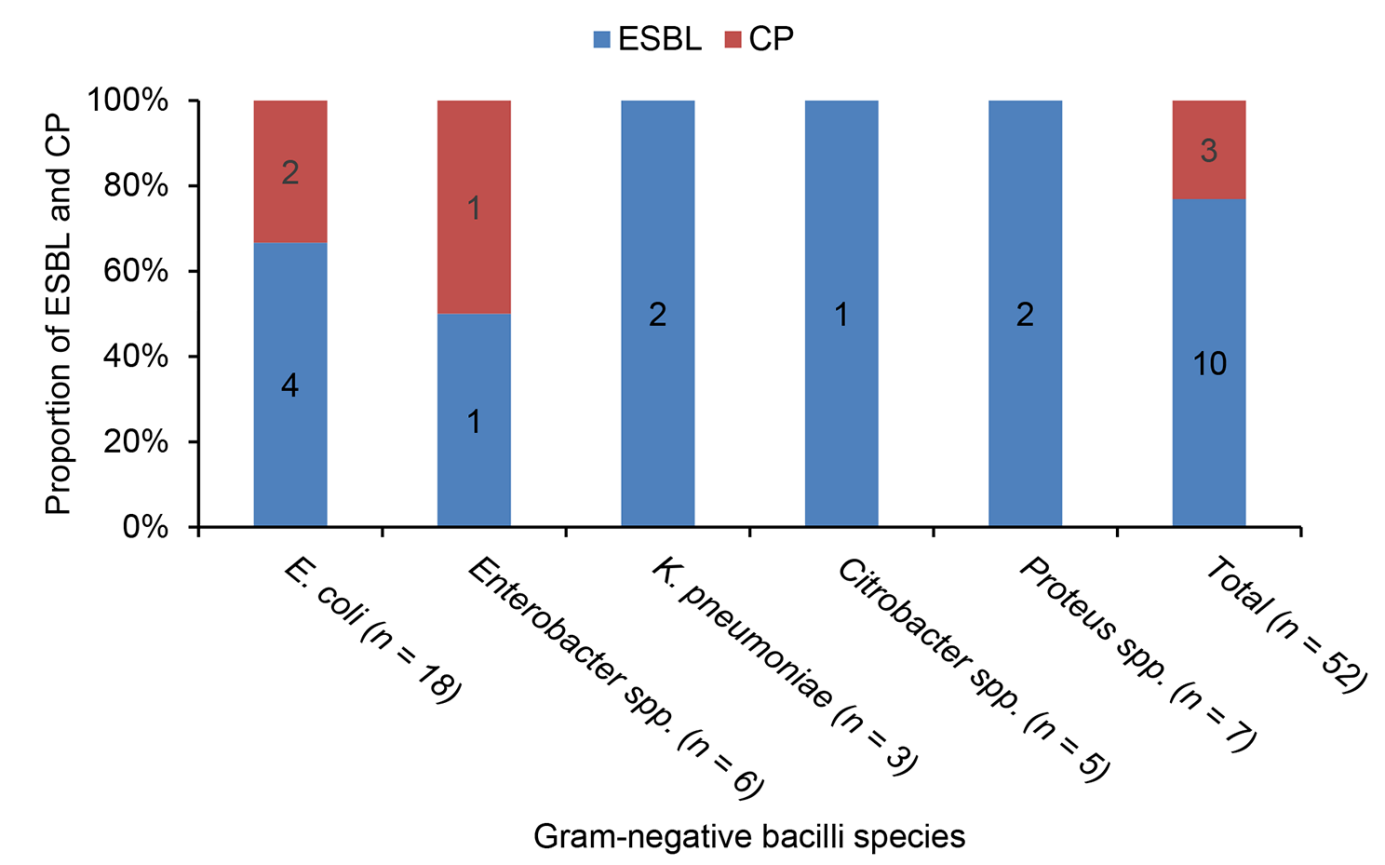
Per hospitals extended-spectrum beta-lactamase and carbapenemase production proportions among Gram-negative bacilli isolates ESBL; Extended-spectrum beta-lactamase; CP, Carbapenemase

Out of 52 GNBs, 10 (19.2%) were considered identified as ESBL producers (Fig. 2). The rate of ESBL expression was 23.3% in FHCSH (7/30) and 13.6% in TGSH (3/22). The proportion of ESBL expression was 22.2% (4/18) in *E. coli*. Similarly, 2 out of 3 *K. pneumoniae* isolates (66.7%) and 2 out of 7 *Proteus* species isolates (28.5%) were ESBL producers (Fig. 2).

Three out of the 52 GNB isolates (5.8%) were considered identified as carbapenemase producers. Of them, 2 were *E. coli* (11.1%), and 1 isolate was an *Enterobacter* species (Fig. 2). All carbapenemase-expressing isolates showed non-susceptibility to all six classes of antibiotics tested; they were also confirmed ESBL producers.

### Antibiotic susceptibility profile of GNB isolates

**Fig. 3:**
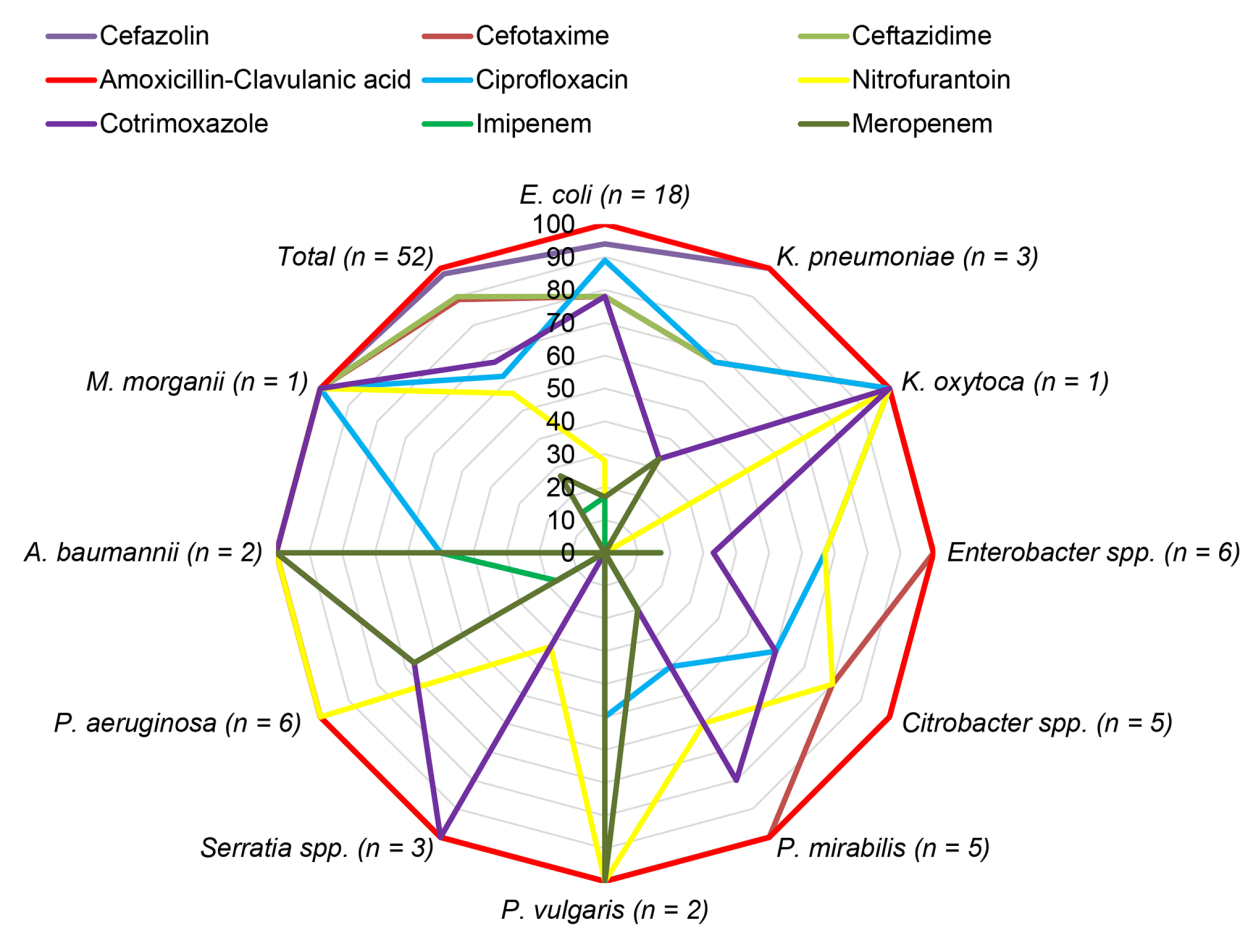
Radar chart showing resistant rates of different Gram-negative bacilli species (*n* = 52) to 9 antibiotics

Of the GNB isolates (*n* = 52) tested for antibiotic susceptibility testing, all were resistant to amoxicillin-clavulanic acid (100%). Moreover, high rates of resistance were recorded for cefazolin (*n* = 51, 98%), ceftazidime (*n* = 47, 90%), and cefotaxime (*n* = 46, 88%). The rate of resistance for meropenem was (*n* = 14, 26.9%) and for imipenem was (*n* = 7, 13%). *E*. *coli* showed a 27.8% rate of resistance to nitrofurantoin (Fig. 3).

In addition to ESBL and carbapenemase, the MDR profiles of all GNBs were evaluated. Forty-five (86.5%) of the 52 GNB isolates were MDR. All *P. aeruginosa*, *Serratia* species, and *A. baumannii* isolates were MDR. In addition, 6 isolates (11.5%) revealed resistance to all six classes of antibiotics tested (Table 2).

**Table 2:** Antibiogram profile of Gram-negative bacilli species isolated from patients with catheter associated urinary tract infections (*n* = 52)

GNB species	Resisted antibiotic combinations	Antibiotic classes	Frequency	Overall MDR N (%)
*E. coli* (*n* = 18)	CZL, CXT, CAZ, AMC, NIT, CIP, COT, MER, IMP	6	3	15 (83.3)
	CZL, CXT, CAZ, AMC, NIT, CIP, COT	5	1	
	CZL, CXT, CAZ, AMC, NIT, CIP	4	1	
	CZL, CXT, CAZ, AMC, CIP, COT	4	6	
	CZL, CXT, CAZ, AMC, COT	3	1	
	CZL, CXT, CAZ, AMC, CIP	3	2	
	AMC, CIP, COT	3	1	
*Enterobacter* species (*n* = 6)	CZL, CXT, CAZ, AMC, NIT, CIP, COT, MER, IMP	6	1	5 (83.3)
	CZL, CXT, CAZ, AMC, NIT, CIP	4	2	
	CZL, CXT, CAZ, AMC, NIT	3	1	
	CZL, CXT, CAZ, AMC, CIP, COT,	4	1	
*K. pneumoniae* (*n* = 3)	CZL, CXT, CAZ, AMC, CIP, COT, MER	5	1	2 (66.7)
	CZL, CXT, CAZ, AMC, CIP	3	1	
*K. oxytoca* (*n* = 1)	CZL, CXT, CAZ, AMC, NIT, CIP, COT	5	1	1 (100)
*Citrobacter* species *(n* = 5)	CZL, CXT, CAZ, AMC, NIT, CIP, COT	5	2	4 (80)
	CZL, CXT, CAZ, AMC, NIT, CIP	4	2	
*P. aeruginosa* (*n* = 6)	CZL, CXT, CAZ, AMC, NIT, COT	4	3	6 (100)
	CZL, CXT, CAZ, AMC, NIT	3	2	
	CZL, CXT, CAZ, AMC, NIT, CIP, COT, MER, IMP	6	1	
*A. baumannii* (*n* = 2)	CZL, CXT, CAZ, AMC, NIT, CIP, COT, MER, IMP	6	1	2 (100)
	CZL, CXT, CAZ, AMC, NIT, COT	4	1	
*Seratia* species (*n* = 3)	CZL, CXT, CAZ, AMC, NIT, CIP, COT	5	1	3 (100)
	CZL, CXT, CAZ, AMC, CIP, COT	4	2	
*M. morganii* (*n* = 1)	CZL, CXT, CAZ, AMC, NIT, CIP, COT	5	1	1 (100)
*Proteus* species (*n* = 7)	CZL, CXT, CAZ, AMC, NIT, CIP, MER	4	1	6 (85.7)
	CZL, CXT, CAZ, AMC, NIT, CIP, COT	5	1	
	CZL, CXT, CAZ, AMC, NIT	4	1	
	CZL, CXT, CAZ, AMC, NIT, CIP, MER	5	2	
	CZL, CXT, CAZ, AMC, COT,	3	1	
**Total**			**45**	**45 (86.5)**

**Key**: CZL: cefazolin, CXT: cefotaxime, CAZ: ceftazidime, AMC: Amoxicillin-clavulanic acid, NIT: nitrofurantoin, CIP: ciprofloxacin, COT: cotrimoxazole, MER: meropenem, IMP: imipenem

### Multivariable analysis of factors associated with CAUTIs

Based on the multivariable analysis, days of catheterization, days of hospitalization, and patient ward were significantly associated with CAUTI. Patients who had catheterization for more than 10 days had an eleven-fold greater chance of developing CAUTI compared to those who had catheterization for less than five days (AOR = 10.6, 95% CI: 1.8–62.1). Similarly, patients hospitalized for more than 10 days had an eight-fold greater likelihood of experiencing CAUTI compared to those with hospital stays below five days (AOR = 8.1, 95% CI: 2.4–27.2). Patients admitted to surgical wards had a 2.8 times higher risk of developing CAUTI (AOR = 2.8, 95% CI 1-7.5) compared to those admitted to medical wards. Likewise, patients admitted to gynecology wards had a 4.3 times higher risk of CAUTI than those admitted to medical wards (AOR = 4.3, 95% CI: 1–17) (Table 3).

**Table 3:** Multivariable analysis of factors associated with catheter associated urinary tract infection

Variables	Confirmed CAUTIs		COR (95% CI)	AOR (95% CI)	*p*-value
	Present	Not present			
**Gender**					
Female	21	119	1.4 (0.7–2.7)*	1.3 (0.6–2.8)	0.54
Male	30	193	1	1	1
**Residence**					
Rural	18	154	2.1 (1.1–4.1)**	1.5 (0.7–3.4)	0.27
Urban	33	158	1	1	1
**Occupation**					
Student	5	25	0.9 (0.3–3.2)*	1.5 (0.2–9.3)	0.662
Private worker	38	224	1.2 (0.5–2.8)*	1.5 (0.5–4.3)	0.449
Government employee	8	62	1	1	1
**Level of education**					
No read and write	18	93	2.4 (0.7–7.7)*	2.2 (0.5–9.2)	0.276
Primary education	21	106	2.9 (0.9–9.4)*	2.9 (0.7–11.1)	0.131
Secondary education	8	62	1.9 (0.5–7.1)*	1.6 (0.4–6.9)	0.503
Diploma and above	4	51	1	1	1
**Patient ward**					
Medical	7	107	1	1	1
Surgical	27	108	4.2 (1.7–1.4)**	3.3 (1.1–9.4)	0.027
ICU	12	63	2.1 (0.7–5.8)*	2.6 (0.9–7.6)	0.083
Gynecology	5	34	4.4 (1.1–16.8)**	4.6 (1.1–19.5)	0.040
**History of hospitalization**					
Yes	19	151	1.6 (0.8–3.1)*	1.2 (0.6–2.7)	0.604
No	32	161	1	1	1
**History of catheterization**					
Yes	17	56	2.6 (1.3–5.3)**	1.6 (0.6–4)	0.331
No	34	256	1	1	1
**Days of catheterization**					
< 5	14	165	1	1	1
5–7	22	120	1.7 (0.8–3.6)*	1.9 (0.8–4.2)	0.130
8–10	11	24	3.9 (1.5–10.2)**	4.3 (1.4–12.6)	0.008
> 10	4	3	7 (1.4–35)	10.6 (1.8–62.1)	0.009
**Days of hospitalization**					
< 5	11	147	1	1	1
5–7	21	118	1.8 (0.8–4)*	2 (0.9–4.6)	0.105
8–10	10	36	3.3 (1.2–9)**	3.3 (1.2–9.1)	0.025
> 10	9	11	6.6 (2.1–20.9)**	8.1 (2.4–27.2)	0.001
**Presence of underlying disease**					
Yes	38	149	0.9 (0.4–1.9)*	2.9 (0.2–43.6)	0.439
No	13	163	1	1	1

Key: CAUTIs (Catheter Associated Urinary Tract Infections), COR (Crud Odds Ratio), AOR (Adjusted Odds Ratio), CI (Confidence Interval), ICU (Intensive Care Unit), 1 (Reference Category), **0.05 < *p*-value > 0.001,*0.2 ≤ *p*-value > 0.05

## Discussion

This study contributed to the knowledge of the incidence of CAUTI caused by GNB and their ESBL and carbapenemase production among hospitalized patients in Northwest Ethiopia. The incidence of CAUTIs caused by GNB (27.8 per 1000 catheter days) in this study was higher than similar reports from various WHO regions in previous years, which ranged from 4.4 to 14.71 per 1000 catheter days [5]. This shows health care-associated infections (HAIs) are a significant burden in the study area and calls for immediate attention to infection control measures and antibiotic stewardship. Further regional studies are crucial to estimating the incidence rate of CAUTI throughout the country.

The incidence rate of CAUTI caused by GNB in the present study (27.8) is consistent with the CAUTI incidence reported from southern Ethiopia (28.1) [6]. However, the incidence of CAUTI reported in our study is higher than reported in Sudan (16.4%) [37], Sierra Leone (14.8%) [38] and other African countries (15.7–16.1) [39, 40]. It is also higher than the CAUTI incidence rates reported from Southeast Asia (14.71), Eastern Mediterranean (9.96), Europe (9.5), Western Pacific (7.18), United States (4.4) [5], and worldwide (12.5–21.9) [41, 42], including Iran (21.9) [10]. The higher incidence rate of CAUTI in the present study compared to the studies mentioned above might be due to several factors. For instance, poor adherence to infection prevention and control procedures and overcrowding of hospital wards might have contributed to the increased rate of CAUTI [43]. In addition, poor adherence to the crucial timing for patient handling and sterile medical equipment in hospital wards could also be linked to the increased rate of CAUTI [44, 45]. Across the different studies cited, a varying socioeconomic or medical background of the respective study participants could contribute to differences in incidence [45, 46].

*E*. *coli*, followed by *Proteus* spp. and *P*. *aeruginosa*, were the most common GNB associated with CAUTI in this study. The predominance of *E*. *coli* and *P*. *aeruginosa* in the present study is consistent with previous studies in southern Ethiopia [6, 13], Sierra Leone [38], Iran [10], and Nepal [42]. *Proteus* spp. is the second leading cause of CAUTI in the present study, while *Klebsiella* spp. was the second leading cause in the above-mentioned studies. This variation could be due to differences in colonizing pathogens, infection prevention and control measures, and the environment.

The ESBL expression among clinical isolates of Gram-negative bacilli is a persistent problem as previous studies in Ethiopia and other African countries reported a high rates of ESBL production [23, 24, 38]. The proportion of ESBL expression among GNB isolates (19.2%) in the present study is lower than what was reported in central Ethiopia (61.7%) [22]. However, it is consistent with a study from Uganda (21.7%) [47]. The possible reasons for the variation in the prevalence of ESBL-expressing GNB between the two studies in Ethiopia could be due to differences in: antibiotic prescription practices, infection control measures, age, comorbidities and overall health status of the patients and selective pressure both at the individual patient level and within healthcare settings [48].

*Klebsiella pneumoniae* and *E. coli* were the main ESBL producers, which is consistent with findings from previous studies [22–24, 47, 49]. This is because *E*. *coli* and *K*. *pneumonia*e are highly prevalent in healthcare settings and are the most commonly encountered bacteria in UTIs.

Apart from the report on carbapenemase prevalence in GNB isolated from urine samples but not from CAUTIs in previous Ethiopian studies [20, 22, 23], there was no report on the status of carbapenemase production in GNB isolated from CAUTIs. Although the present study included only new cases of catheterized patients, while the studies mentioned above [20, 22, 23] assessed both new and chronic cases of UTI and employed a modified carbapenem inactivation test, the rate of carbapenemase-producing GNB (5.8%) in patients with CAUTI in the present study fell within the spectrum of rates (ranging from 2.73 to 15.2%) reported in previous studies mentioned above [20, 22, 23]. This showed carbapenemase production in Enterobacteriaceae is a consistent problem in Ethiopia. *E. coli* (11.1%) and *Enterobacter* species (16.7%) were the only carbapenemase-producing isolates identified in the present study. This is in agreement with the carbapenemase-producing isolates reported in the study conducted in northwest Ethiopia [20].

A high level of antimicrobial resistance was observed in this study. Specifically, to amoxicillin-clavulanic acid, cefazolin, cefotaxime, and ceftazidime. Uncontrolled and repeated use of antibiotics in the study area might be the cause of the high level of resistance to third-generation cephalosporins. These antibiotics are the most frequently prescribed antibiotics in the study area, and their extensive usage might contribute to the development and spread of resistance among GNB.

The level of GNB resistance to third-generation cephalosporins in this study was notably higher than the rates reported for GNB isolated from different clinical specimens in FHCSH, Ethiopia, which showed resistance rates of 58% for cefotaxime and 57% for ceftazidime [24]. This variation may be because the present study exclusively focused on UTIs in catheterized patients, and these patients were more likely to have been colonized by MDR organisms prior to the infection. GNB from this specific group might have an increased likelihood of experiencing antibiotic resistance by the formation of catheter-assisted biofilms [50].

Multi-drug resistance among GNB poses a significant concern in Ethiopia, with a high rate of 86.5% observed in patients with CAUTI in this study. Another investigation in northwest Ethiopia reported a similar MDR rate of 87.4% among Enterobacteriaceae isolates from urine samples [20]. The high MDR rate in Enterobacterales and non-fermenting GNB isolates in this study, despite lower percentages of ESBL and carbapenemase producers, can be explained by several factors. Beyond ESBL and carbapenemase production, diverse resistance mechanisms exist. The GNB isoaltes in this study may harbor additional resistant genes such as Amp C beta-lactamase, efflux pump, and alterations in membrane permeability, contributing to the overall high MDR profile. Widespread antibiotic use in healthcare settings in Ethiopia can exert selective pressure, leading to the emergence of MDR phenotype that may not necessarily express ESBL or carbapenemase enzyme [51]. Strains with both ESBL and carbapenemase production would be resistant to beta-lactams and other antibiotic classes. For instance, all carbapenemase-producing isolates of this study exhibited non-susceptibility to all six classes of antibiotics tested and were confirmed ESBL producers.

Factors such as inadequate infection control measures, lack of surveillance on antimicrobial resistance, horizontal genes transfer, and clonal spread can contribute to the high MDR rates, even in the absence of ESBL or carbapenemase production [53]. In addition, there might be novel resistance mechanisms.

Prolonged catheterization is a significant predictor variable associated with CAUTI. This is consistent with similar studies conducted in Ethiopia [6], Brazil [52] and Italy [41]. The increased risk of CAUTI among patients with prolonged catheterization might be linked to the fact that prolonged catheterization and increased hospital stays create a conducive environment for the ascending of enteric pathogens into the bladder through the catheter. The continuous presence of a catheter in the urinary system can facilitate the colonization and proliferation of the bacteria, increasing the likelihood of infection. Length of hospital stay was also significantly associated with CAUTI in the present study, which is in line with findings from Egypt [39], USA [53]. During extended hospitalization, patients are more exposed to nosocomial pathogens and have developed resistance to many antibiotics [54]. However, the presence or absence of MDR pathogen colonization before the current hospital admissions was not differentiated, as we did not perform any bacteriological tests for any screening for enteric MDR colonization. Therefore, the interpretation of these findings needs caution. Furthermore, patients from surgical wards had the highest risk of developing CAUTI in the current study. This might be due to the contamination of catheters from postoperative infected wounds and spread to the urinary tract. In addition, patients from surgical wards have slight longer catheterization days and inpatient days compared to patients in other wards.

The study was conducted in two specialized public hospitals in Northwest Ethiopia and included a relatively large sample size of 363 patients and followed over time for the assessment of CAUTIs using a longitudinal study design which enhances the representativeness, reliability, and generalizability of the results at the regional scenario. The study used chromogenic medium to investigate the production of ESBL and carbapenemase among patients with CAUTI, providing valuable insights into the burden of drug resistant pathogens in Ethiopia. However, chromogenic ESBL/carbapenemase media do not allow for direct proof of ESBL/Carbapenemase expression, and is not 100% specific. Because of the unavailability of VITEK or Phoenix in the study area, the likely ESBL and carbapenemase producing isolates were not retested by these complementary methods. In addition, due to the absence of PCR and sequencing techniques in the study area, the mechanisms of the high rate of MDR phenotypes were not further tested, therefore, the results interpreted and discussed with caution. Furthermore, the study did not test the susceptibility of GNB isolates to colistin due to the unavailability of this antibiotic in the market.

## Conclusions

Higher incidence of CAUTIs due to Gram-negative bacilli, most of which are MDR is found in Northwest Ethiopia compared to previous sub-Saharan African countries reports. *E*. *coli*, *Proteus* spp. and *P*. aeruginosa ranked first, second, and third, respectively in CAUTI patients. These isolates were also the major MDR and showed a super high rate of resistance to amoxicillin-clavulanic and 3rd generation cephalosporins which showed empirical treatment with these substances which is very common in Ethiopia, is virtually ineffective in patients with suspected GNB infection. The considered, confirmed ESBL and carbapenemase producing GNB isolates in CAUTI patients highlighting the consistent importance of monitoring and controlling the spread of drug-resistant bacteria. Longer days of catheterization and longer days of inpatient stay are the factors associated with CAUTI, suggesting the need for improved infection prevention and control practices in healthcare settings. Therefore, regular, and multicenter studies are crucial to guide empirical treatment, improving patient outcomes and reducing the burden of CAUTIs and MDR in the country.

## Data Availability

All data generated or analyzed during this study are included in this manuscript.

## Abbreviations

ATCC: American Type Culture Collection
CAUTIs: Catheter associated Urinary tract infections
CLSI: Clinical and Laboratory Standards Institute
ESBL: Extended-spectrum Beta-lactamase
FCSH: Felege Hiwot Comprehensive Specialized Hospital
GNB: Gram-negative bacilli
MDR: Multidrug-resistant
TGSH: Tibebe Ghion Specialized Hospital
UTIs: Urinary tract infections
